# Mast cell activation in the acupoint is important for the electroacupuncture effect against pituitrin-induced bradycardia in rabbits

**DOI:** 10.1038/s41598-017-08855-5

**Published:** 2017-08-22

**Authors:** Haining Zhu, Xuezhi Wang, Meng Huang, Yi Jing, Di Zhang, Guanghong Ding

**Affiliations:** 1 Department of Aeronautics and Astronautics, Fudan University, Shanghai, 200433 China; 2 Shanghai Key Laboratory of Acupuncture Mechanism and Acupoint Function, Fudan University, Shanghai, 200433 China; 3 Shanghai Research Center for Acupuncture and Meridian, Shanghai, 201203 China

## Abstract

This research was conducted to verify the structural and functional characteristics of mast cells in the electroacupuncture (EA) effects on bradycardia. First, we examined the mast cell density at PC 6, adjacent acupoint LU 7, and a non-acupoint. We tested the effects of EA at PC 6 on heart rate (HR) and blood pressure (BP) in rabbits with pituitrin-induced bradycardia. We also injected sodium cromolyn (Cro), a mast cell membrane stabilizer, at PC 6 30 min before EA to investigate if it affected the EA effects. The results showed that in both PC 6 and LU 7, the mast cell densities were higher than in the non-acupoint (*P* < 0.05). EA could induce mast cell degranulation at PC 6, which could be suppressed by sodium cromolyn (*P* < 0.05). EA improved HR, though the change was relatively small in the initial stage with a significant change at 35 min after modelling (*P* < 0.05). BP significantly improved at 10 min after the onset of pituitrin-induced bradycardia (*P* < 0.05). The EA effects on both HR and BP were suppressed by sodium cromolyn (*P* < 0.05). Therefore, we concluded that mast cells in the acupoint are important for the EA effects against pituitrin-induced bradycardia in rabbits.

## Introduction

The mechanism of the activation process of an acupoint during acupuncture is the key to understanding acupuncture’s effects. During acupuncture, mechanical or electrical stimuli are applied to acupoints by acupuncture needles to generate effective biological signals. It has been reported several times that mast cell densities at acupoints are higher than in adjacent areas^1^. In rats, both the connective tissue and the muscle at ST36 have higher mast cell densities than do tissues in nearby areas^2^. Even in the primo vascular system (PVS), which was proposed as the anatomical structure of the acupuncture points and meridians, also has a high density of mast cells^3^. These preliminary research studies have prompted an interesting question: Does the characteristic feature of mast cell densities exist extensively in the skin? Conversely, is the specific trait only observed in the ST 36 acupoint? To investigate mast cell densities in the skin, we examined the dermal tissue of PC 6, an adjacent acupoint, LU 7, and a non-acupoint area at the midpoint between these two acupoints to attempt to achieve a more universal conclusion.

In our previous studies, we provided strong evidence to show that mast cells in an acupoint are important for EA-induced analgesia in adjuvant-induced arthritis rats. The local administration of either sodium cromolyn or the histamine H1 antagonist clemastine could abolish the analgesic effects of manual acupuncture^4, 5^. Shi *et al*. found that mast cells behaved in a same way in rats with colitis during moxibustion^6^. Moreover, Wang showed that different degranulation ratios of mast cells during thermal stimulation correspond to cardiac function changes in a bradycardia rat model^7^. These studies suggest that the function of mast cells may be universal for the activation of an acupoint regardless of the acupoint or the treatment. Many researchers have also investigated the acupuncture regulation effect on heart rate and blood pressure. Bäcker found that diverse modes of manual acupuncture stimulation have different effects on the modulation of heart rate and blood pressure in human subjects^8^. Frank verified that 24-hour ambulatory blood pressures can be significantly lowered after 6 weeks of acupuncture treatment^9^. Friedemann also found that mean arterial blood pressure decreases during acupuncture treatment in anaesthetized rats^10^. Ohsawa and Uchida showed that depressor responses of arterial pressure and reflex inhibition of the heart rate can be elicited by acupuncture-like stimulation^11, 12^. In addition to manual acupuncture, electrical acupuncture is also considered an effective treatment for various diseases. The electrical stimulation of four specific acupuncture points could provoke a significant, immediate post-stimulation reduction in diastolic blood pressure in hypertensive subjects^13^. Stephanie also demonstrated that electroacupuncture modulation of elevated blood pressure responses works via the paraventricular nucleus (PVN) and its projection to the rostral ventrolateral medulla (rVLM) through a PVN opioid mechanism^14^. To date, researchers have conducted many investigations to determine the relationship between acupuncture and mast cells, and several others have discovered the acupuncture effect on the modulation of HR and BP. However, few studies have attempted to identify the relationship between mast cells and the indexes of HR and BP via acupuncture or explore the underlying mechanism. Therefore, we tested the effects of EA on heart rate (HR) and blood pressure (BP) in rabbits with pituitrin-induced bradycardia. Furthermore, we investigated whether an injection of sodium cromolyn, a mast cell membrane stabilizer, into acupoints affects the EA effects. This research may improve our understanding of the function of mast cells in acupoint activation.

## Results

### Differences in mast cells densities between acupoints and non-acupoints

**Figure 1:**
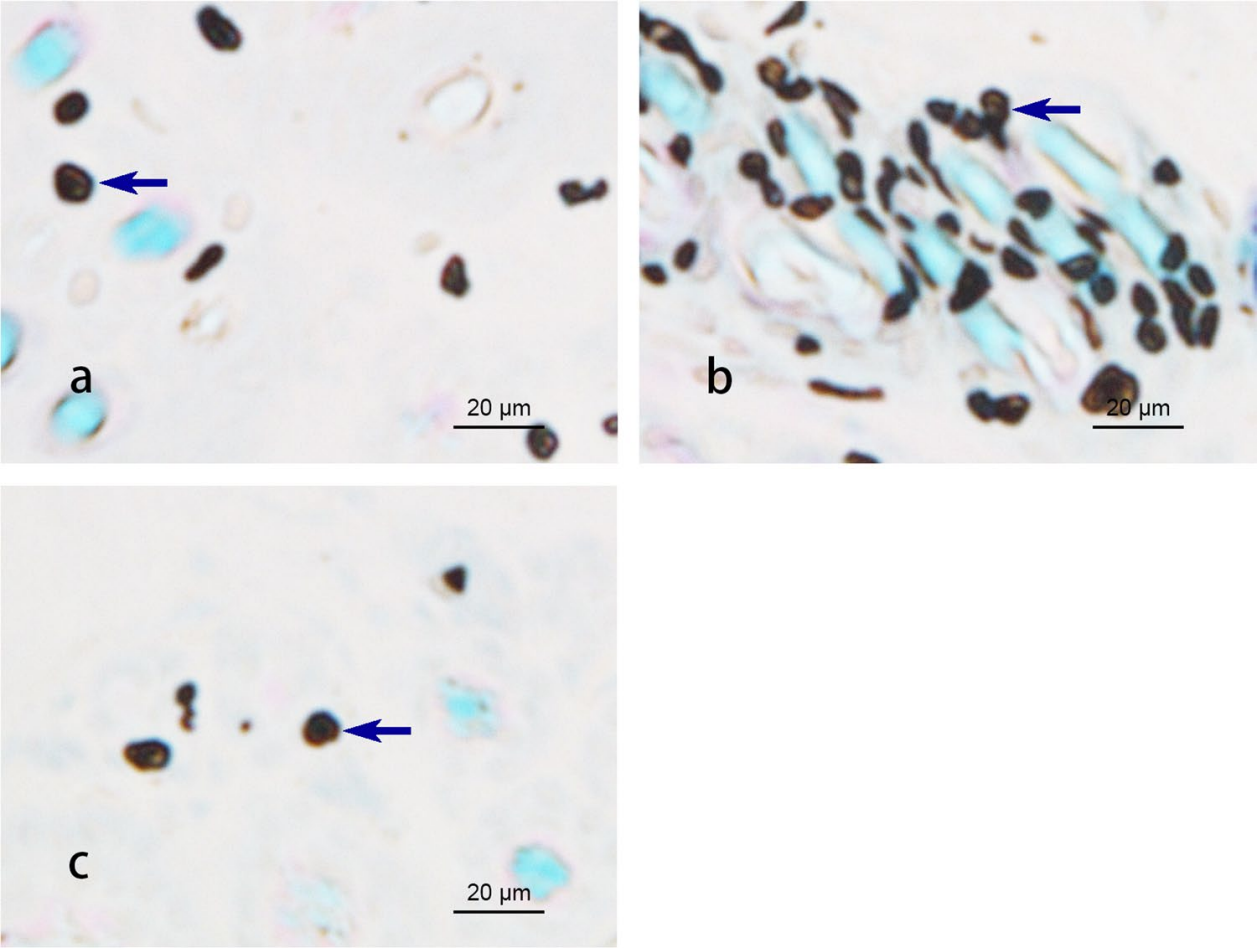
Densities of mast cells in the dermal tissue at PC 6, LU 7 and the non-acupoint (×400).  Indicates mast cells.

**Figure 2:**
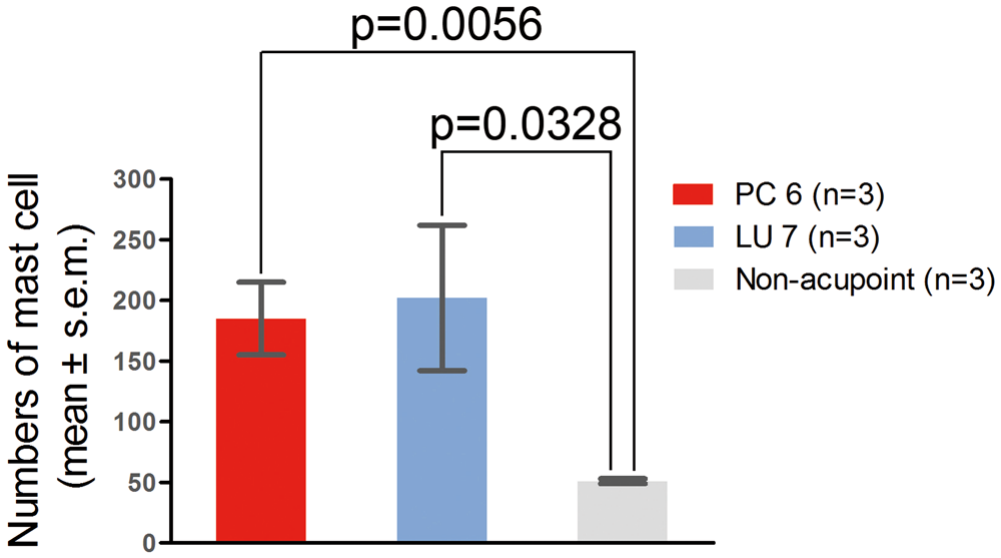
Numbers of mast cells in PC 6, LU 7 and the non-acupoint (n = 3).

To compare mast cell densities in acupoints and non-acupoints, the numbers of mast cells in PC 6, LU 7, and non-acupoint dermal tissue in healthy animals were counted. Figure 1 shows the mast cells after combined staining with toluidine blue and safranine O. The cells appeared dark brown, had diameters of approximately 10 μm and were distributed randomly in the tissues. In both acupoints, more mast cells were found than in the non-acupoint. No significant difference was observed between PC 6 and LU 7 in terms of their numbers of mast cells. However, mast cell densities in both acupoints were significantly higher than in the non-acupoint (Fig. 2, P < 0.05).

### Degranulation of mast cells after EA

**Figure 3:**
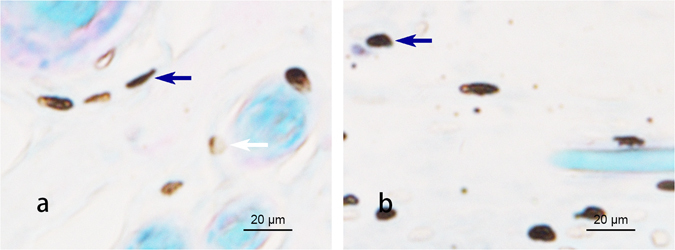
Degranulation of the mast cells in PC 6 after EA. (×400). The mast cells were stained with toluidine blue combined with safranine O. Note that after EA, the mast cells in PC 6 were degranulated, as shown by . In the Cro + EA group, most mast cells were stable without granules released, as shown by .

**Figure 4:**
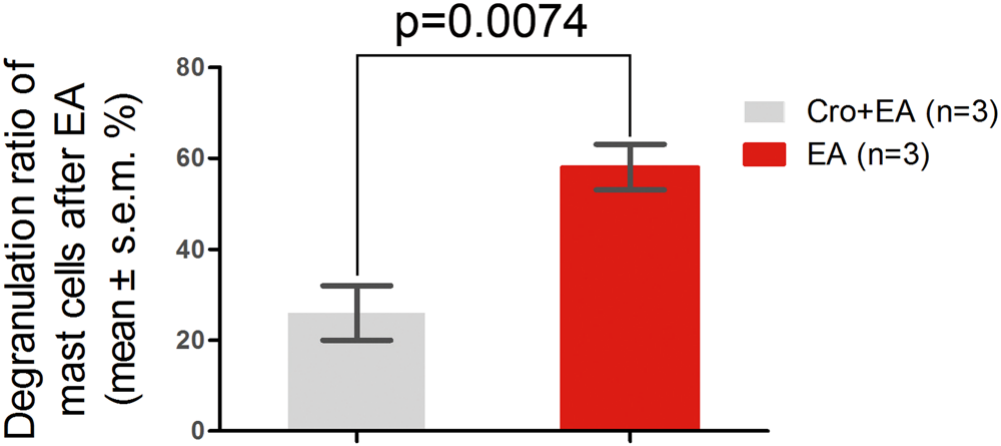
Degranulation ratios of mast cells in PC 6 after EA vs. Cro + EA (n = 3).

To examine the relationship between EA effects and the activation of local mast cells, we injected sodium cromolyn into the acupoints prior to EA treatment. The results show that after EA, the mast cells in PC 6 degranulated (see below Fig. 3, white arrow). In contrast, in the Cro + EA group, most of the mast cells maintained smooth surfaces and few granules were released. The average degranulation ratio of the EA group was approximately 2 times greater than the Cro + EA group (see below Fig. 4, P < 0.05).

### EA effects on heart rate and blood pressure in pituitrin-induced bradycardia

**Figure 5:**
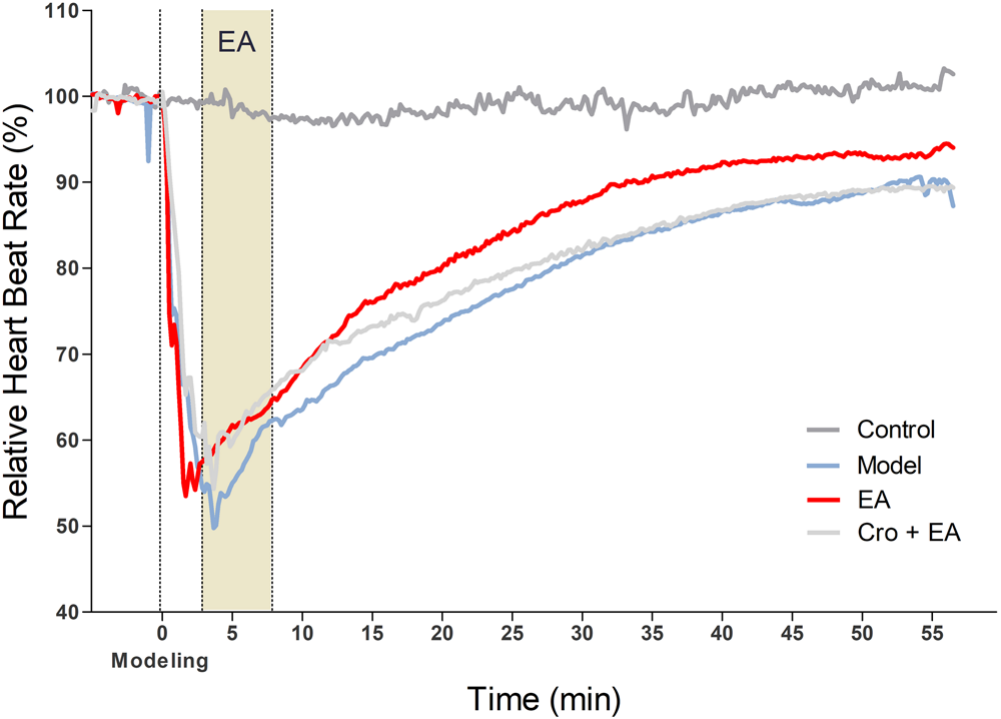
Average relative heart rate in different groups (n = 5–6). The heart rate was recorded in each animal by ECG. The average of every 10 s was calculated. The average relative heart rate in each group is shown with different colours. Intravenous pituitrin was injected to induce bradycardia 5 min after recording (indicated as “Modelling”, dashed line). EA was applied at PC 6 at 3 min after modelling (shadow area).

**Figure 6:**
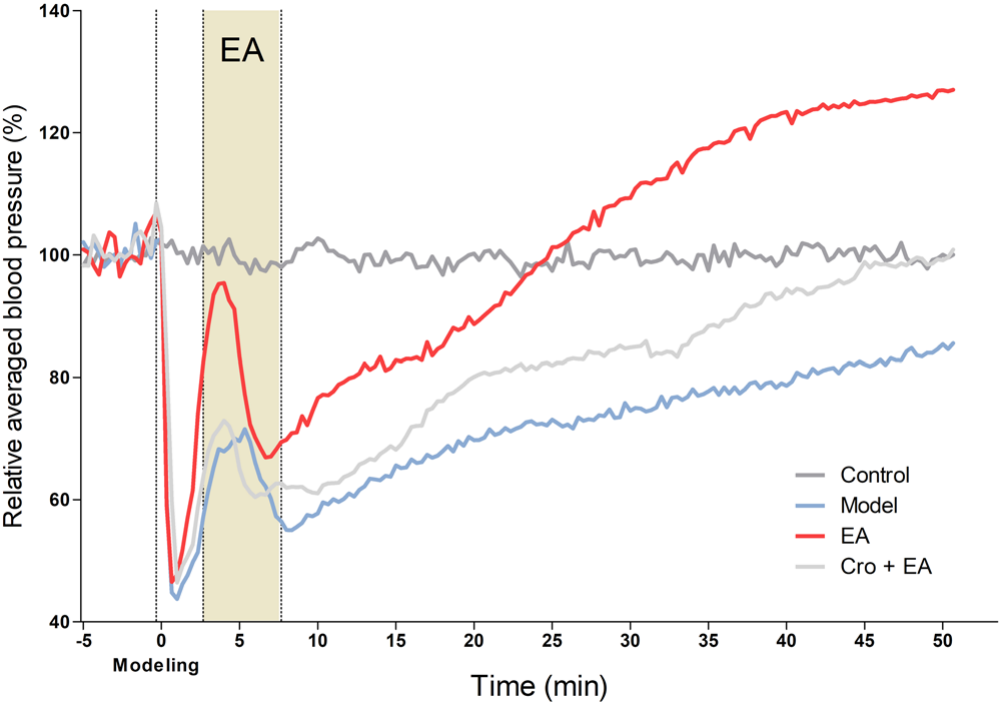
Relative blood pressures among different groups (n = 5–6). Blood pressure was measured at the left carotid artery. The average of 50 beats was calculated for every 20 s. The average relative blood pressures of each group are shown with different colours. An intravenous injection of pituitrin was applied to induce bradycardia 5 min after recording, indicated as “Modelling” with a dashed line. EA was applied at PC 6 at 3 min after modelling (shadow area).

To investigate the EA effects on bradycardia, we recorded electrocardiograms (ECG) during EA at PC 6. The results show that during the whole recording, the HR of the control group remained stable. An intravenous injection of pituitrin largely reduced the HR. In less than 5 min, all groups reached the minimum HR, which was approximately 50% of the control level. Next, the HR gradually recovered and returned to approximately 90% of the control level at the end of recording (Fig. 5). We compared the HRs among the groups every 5 min. The HR of the pituitrin group was significantly lower than that of the control group (Table 1, P < 0.05). EA slightly improved the recovery of the HR, with a significant difference at 35 min between the EA and non-EA groups (Table 1, P < 0.05). The HR of the Cro + EA group was significantly lower at 35 min and 40 min compared to the EA group without Cro (Table 1, P < 0.05). We also recorded BP in the left carotid artery during the experiment. The results show that during the whole recording, the BP of the control group remained stable. In all bradycardia groups, the BP dropped immediately after the intravenous injection of pituitrin, reaching the first minimum BP (approximately 50% of the control) in approximately 3 min. The BPs in these groups then went through a quick recovery phase and then dropped to a second minimum value in approximately 10 min. The BPs in these groups continuously increased after 10 min (Fig. 6). We compared the BPs between the groups every 5 min. In the Model group, the BP decreased significantly after 10 min (Table 2, P < 0.05). After EA at PC 6, the recovery of BP in the EA group was significant compared with the Model group (Table 2, P < 0.05). The recovery of BP in the Cro + EA group was significantly suppressed compared with the EA group after 25 min (Table 2, P < 0.05). Therefore, EA at PC 6 could improve the recovery of blood pressure in bradycardic animals, which could be suppressed by the local administration of sodium cromolyn before EA.

**Table 1:** Comparison of relative heart rates among the groups (AVG ± SEM).

Time	Control	Model	EA	EA + Cro
(min)	N = 5	N = 5	N = 5	N = 6
−5	100.2 ± 0.41	100.33 ± 0.14	100.21 ± 0.09	98.32 ± 2.18
5	97.56 ± 1.47	55.01 ± 7.28*	61.75 ± 4.73	59.67 ± 4.35
10	97.46 ± 0.73	63.57 ± 5.26*	68.36 ± 4.81	68.09 ± 3.52
15	97.06 ± 0.48	69.57 ± 2.69*	75.99 ± 4.42	73.26 ± 4.28
20	97.94 ± 0.96	73.69 ± 3.39*	80.19 ± 3.13	76.23 ± 4.19
25	99.62 ± 1.2	77.56 ± 3.61*	84.33 ± 2.25	79.72 ± 3.73
30	99.12 ± 1.76	81.49 ± 3.6*	87.64 ± 1.7	81.99 ± 2.96
35	98.6 ± 1.14	84.21 ± 2.97*	90.75 ± 1.63^⚫^	84.56 ± 2.33^○^
40	99.32 ± 2.11	86.59 ± 2.45*	92.33 ± 2	86.68 ± 1.72^○^
45	100.72 ± 2.55	87.67 ± 2.01*	92.58 ± 1.98	88.27 ± 1.63
50	101.1 ± 3.62	88.7 ± 1.63*	93.18 ± 2.02	88.95 ± 1.54
55	100.72 ± 3.11	90.31 ± 1.74*	93.45 ± 1.74	89.51 ± 1.74

^*^P < 0.05, Control vs. Model; ^●^P < 0.05, Model vs. EA; ^○^P < 0.05, EA + Cro vs. EA.

**Table 2:** Comparison of the relative blood pressures among the groups (AVG ± SEM, n = 5–6).

Time	Control	Model	EA	Cro + EA
(min)	N = 5	N = 5	N = 5	N = 6
−5	98.25 ± 1.31	102.09 ± 1.65	100.9 ± 2.46	98.29 ± 0.63
5	98.7 ± 0.83	69.59 ± 14.39	83.36 ± 14.77	65.02 ± 10.35
10	102.7 ± 2.43	57.77 ± 2.74*	76.64 ± 5.17^●^	61.04 ± 5.92^○^
15	99.21 ± 0.8	65.61 ± 5.17*	82.8 ± 6.47^●^	68.17 ± 5.55
20	100.22 ± 1.71	69.73 ± 8.5*	88.66 ± 8.03	80.08 ± 4.37^○^
25	97.39 ± 0.28	73.09 ± 9.64*	101.26 ± 8.07^●^	81.93 ± 4.52^○^
30	100.75 ± 0.79	74.56 ± 8.91*	109.3 ± 8.3^●^	84.87 ± 6.25^○^
35	97.43 ± 0.71	77.74 ± 9.07*	117.42 ± 8.28^●^	88.37 ± 7.88^○^
40	101.31 ± 2.03	78.64 ± 9.21*	123.41 ± 8.25^●^	94.41 ± 9.1^○^
45	101.21 ± 2.24	82.11 ± 9.95	124.74 ± 8.46^●^	98.82 ± 9.12^○^
50	100.03 ± 0.36	85.42 ± 10.75	126.96 ± 8.64^●^	99.1 ± 8.44^○^

^*^P < 0.05, Control vs. Model; ●P < 0.05, Model vs. EA; ^○^P < 0.05, Cro + EA vs. EA.

## Discussion

Our studies show that (1) mast cell densities in PC 6 and LU 7 are significantly higher than in the selected non-acupoint in rabbits; (2) in mast cells, the degranulation ratio in PC 6 after EA is higher than the spontaneous ratio; (3) the intradermal administration of sodium cromolyn prior to EA can suppress mast cells degranulation; (4) electrical stimulation at PC 6 seems to promote the recovery of both HR and BP, which are reduced under pituitrin-induced bradycardia; and (5) the EA regulation effects on both HR and BP could be abolished by the local administration of sodium cromolyn prior to EA. These data suggest that mast cells in PC 6 are involved in the process of acupoint activation and in EA effects.

In the present experiment, we chose PC 6 as the primary acupoint for electro-stimuli manipulation. PC 6 is widely reported to be used in the treatment of heart diseases in articles on traditional Chinese medicine and is also used as the main acupoint to improve heart function performance clinically. LU 7 is an acupoint that is close to PC 6. In traditional Chinese medicine, LU 7 is reported to be used to treat various diseases. We believe that LU 7 is another good acupoint to detect the distribution of mast cells compared to non-acupoints. For the choice of a non-acupoint, we think that it should be close to PC 6 but should also be appropriately far from PC 6 at the same time. Additionally, the non-acupoint should be an appropriate distance away from other acupoints and should not be at any meridian.

Many reports have shown that the number of mast cells in an acupoint is greater than in non-acupoints^15–17^. In this study, we also found that the mast cell densities in PC 6 and LU 7 were higher than in non-acupoints (Fig. 1). In addition to the number of mast cells, the degranulation ratio of mast cells is equally significant during the activation of an acupoint. Previous research studies have verified that the mast cell degranulation ratio after acupuncture is higher than the spontaneous ratio^2, 4, 18, 19^. This phenomenon was also observed in our experiment (Fig. 3) and refers to when the substances inside a mast cell are released to the extracellular environment through the process of degranulation, which has been thought to exert an influence on adjacent neurons^20^. PC 6, the primary acupoint of the Pericardium meridian, exhibits a moderating function to improve heart performance in bradycardia and myocarditis. Stimulation at PC 6 has been shown to enhance heart function in several studies^21–24^. EA was chosen as the treatment method in our experiment. The results show that both heart rate and blood pressure perform better after electrical acupuncture to PC 6 compared with the same stimulation to a non-acupoint (Tables 1 and 2). Nonetheless, the acupuncture regulatory effect could be suppressed by the local administration of sodium cromolyn prior to EA (Tables 1 and 2). Considering that the degranulation ratio of the mast cells in the acupoint was lowered with the injection of sodium cromolyn before EA, we propose that the density and the degranulation ratio of mast cells may be involved in the regulatory acupuncture effect on both HR and BP.

Mechanism research studies on acupuncture have shown that mast cells in acupoints are important functional cells for the activation of an acupoint. During acupuncture, mechanical stimuli can be transmitted to local mast cells through collagen deformation, which induces the degranulation of mast cells^19^. During the degranulation of mast cells, several channels and mechanisms become involved^25, 26^. Mechanical stress can lead to the activation of the TRPV2 channel, one of the channels involved in the degranulation of mast cells. This channel allows Ca^2+^ ions to enter the cell and ultimately induces the degranulation of mast cells^27^. For EA, Zhu *et al*. proved that different frequencies of electric fields can cause changes in the intracellular calcium ion concentration^28^. Mediators released from the degranulation of mast cells, including histamine and adenosine, may play important roles in signal transmission between mast cells and neural cells^18^. Ohsawa proved that the modulation of mean arterial blood pressure induced by acupuncture-like stimulation is a reflex response and that the afferent pathway contains muscle afferents^12^. Uchida showed that the reflex pathway for the modulation of heart rate by acupuncture-like stimulation consists primarily of group IV muscle afferent fibres and that the activity of these fibres can lead to the activation of GABAergic neurons in the brainstem^11^. Additionally, the change in enkephalin in the Rostral Ventral Latermal Medulla may induce the regulation of cardiovascular function via electro-stimulation^29^. The local administration of a histamine H1 antagonist suppresses acupoint activation, suggesting that the histamine released by mast cells in an acupoint acts as a mediator^4^. The direct injection of an adenosine A1 receptor agonist also replicated the analgesic effect of acupuncture since adenosine is a neuromodulator with anti-nociceptive properties^30^. The release of ATP, the metabolic source of adenosine, is also involved in the degranulation of mast cells^31^. Therefore, the degranulation of mast cells is the basis of the influences that we mentioned before. However, sodium cromolyn is known to inhibit histamine release from human lung mast cells^32^. The inhibitory effects of disodium cromoglycate work at the first antigen-dependent and extracelluar Ca^2+^-independent stage of the mast cell activation processes in rats, increasing membrane permeability and causing an influx of extracellular calcium in the second stage prior to the degranulation of mast cells^33^. In addition, sodium cromolycate has been suggested to stabilize neuronal membranes^34^. Further data has shown that neuronal damage may be involved in histamine release, which can be diminished by sodium cromolycate pretreatment^20^. Sodium cromolyn has an adjunctive role in asthma due to its excellent effectiveness, especially in children. It also has therapeutic roles in such diseases as mastocytosis and allergic conjunctivitis^35^.

According to prior studies, mast cells in an acupoint serve as targets of acupuncture under various circumstances. It has been demonstrated that the acupuncture effect on ST 36 can be markedly reduced by the intradermal administration of sodium cromolyn in rats^2, 5, 18^. In rats with colitis, the moxibustion effect was also shown to be suppressed by the same mast cell stabilizing agent^6^.

Considering our results and those from previous research, we can speculate that despite the treatment, disease model or acupoint, mast cells have broad, important roles in the activation of an acupoint. The mast cell may be a universal regulator in the process of transducing physical stimuli to biological acupuncture signals in an acupoint. Former research on animals and clinical studies have suggested that PC 6 is closely related to the regulation of heart rate and blood pressure. Stimulation on PC 6 could trigger effective signals in the CNS. However, the transduction process of physical stimuli into biological signals at an acupoint remains unknown. The differences in acupuncture effects on biological signals and physiological regulations may only exist in the process of the integration of an acupoint’s signals in the central nervous system. The activation process at an acupoint may be universal across different diseases and acupoints. Research on acupuncture effects in the CNS and in targeted organs is important to unveil the mechanisms of specificity in acupuncture treatments for different diseases. Such research could provide theoretical support to clinical studies and improve the understanding of the process of acupoint activation by revealing the relationship between acupuncture and the physiological functions of acupoints.

## Methods and Animals

### Animals

The present study was performed in accordance with the guidelines of the Animal Care and Use Committee of Shanghai Research Center for Acupuncture and Meridian, which are based on the NIH’s Guide for the Care and Use of Laboratory Animals. The experimental protocols were approved by the Animal Care and Use Committee of Shanghai Research Center for Acupuncture and Meridian. Male New Zealand Rabbits (2.0 ± 0.2 kg), from Shanghai Shengwang Experimental Animals Ltd., were housed in cages in a temperature-controlled environment (22–25 °C) with a 12/12-hour light/dark cycle. Food and water were freely available. All animals were handled with care to prevent infection and to minimize stress. The animal experiments were performed between 9 am and 5 pm. For each experimental group, the animals were chosen randomly. All animals were housed for at least 2 days before experimentation.

### HR and BP recording

The animals were anaesthetized with intravenous urethane (200 g/L, 5 ml/kg) and pentobarbital sodium (20 g/L, 1 ml/kg). Next, the hair on each animal’s neck was carefully removed. The animals were placed on their backs during the whole recording. Two unipolar limb leads were employed in electrocardiography (right hind arm “ + ”, left front arm “−”, right front arm “ground”). Blood pressure was assessed at the left carotid artery through intubation. The data was collected using a multichannel physical recording system (Power lab 16/30, AD Instruments) with amplifiers for electrocardiography (Power lab Dual Bio Amp ML135, AD Instruments) and blood pressure (Power Lab Quad Bridge Amp M112L, AD Instruments). All recordings were started 20 min after operation.

### Groups

Twenty-one animals were randomly divided into 4 groups: the Control group (Control) was recorded for 55 min without any treatment. The Model group (Model) was given an intravenous injection of pituitrin (6 units/ml, 2 units/kg) in the ear 5 min after recording. The EA group (EA) was given 5-min EA at PC 6 at 3 min after the injection of pituitrin. The Cromolyn group (Cro + EA) received the intradermal administration of 100 μl of 0.02 g/ml sodium cromolyn (Cromolyn Sodium Salt, Sigma) 30 min before EA. Another 3 animals were used to investigate the mast cells densities in different points.

### Acupoints

PC 6 is designated the point at 1/6 of the anterior forearm length above the rasceta between the ulna and the radius. LU 7 is designated the point at 1/8 of the anterior forearm length above the rasceta along the outside edge of the radius. The non-acupoint is at the midpoint between PC 6 and LU 7. Both acupoints are designated according to their respective anatomical locations on humans.

In the EA and Cro + EA groups, a 0.25 × 13 mm acupuncture needle was inserted into PC 6 at a depth of approximately 2 mm. Twisting and lifting were applied to the needle to achieve “de qi”, which was felt as the needle was grabbed by the tissue. One mm above PC 6, another needle was inserted at a depth of approximately 1 mm. Both needles were then connected to the EA equipment (H.A.N.S electro- acupuncture stimulator, China). We used 2-Hz square waves in this experiment. The current was set to 4 mA.

### Histological study

After HR and BP recordings, the animals were sacrificed using an intravenous injection of air. A dermal tissue sample of 3 × 3 × 3 mm^3^ around PC 6 was carefully dissected. For the mast cells densities investigation, the animals were sacrificed via pentobarbital sodium overdose. Dermal tissues at LU 7, PC 6 and the non-acupoint were dissected in the same way as described above. All tissue samples were immediately soaked in Carnoy’s solution for 20 hours. Paraffin slices of 10-μm thickness were made for approximately every other 0.15 mm on the tissue. The sections were longitudinal to the skin surface. All slices were stained with toluidine blue and safranine O (0.5 mol/L HCL 5 min, 0.5 mg/ml toluidine blue (PH = 0.5) 45 min, 0.5 mol/L HCL 30 s, 0.25 mg/ml safranine O 30 s). The mast cells were counted under a microscope. For each sample, the mast cells in 3 slices were counted and the mast cells in each slice were counted in 3 randomly chosen areas of 0.3 × 0.3 mm^2^. Mast cells with more than three granules released or clear cavities were considered degranulated. The degranulation ratio was calculated as the number of degranulated mast cells divided by the total number of mast cells.

### Data analysis

Lab Chart 7.0 was used for data collection for electrocardiography and blood pressure measurements. The sample rate was 2 k/s. HR and BP were calculated by HRV (heart rate variability) and Blood Pressure modules in Lab Chart 7.0. The average HR for every 10 s and the average BP of 50 beats for every 20 s were then calculated. The relative HR and BP were calculated against the average of the data 5 min before modelling in each animal.

All data were analysed using Excel. The relative HR and BP were tested for homogeneity of variance through an F-test. Depending on the results of the F-test, the relative HR and BP were compared between the groups with a T-test (one-tailed) under either heteroskedasticity or homoscedasticity assumptions. The average number of mast cells and the degranulation ratio were compared between the groups with a T-test (one-tailed).
